# Pico and nanoplankton abundance and carbon stocks along the Brazilian Bight

**DOI:** 10.7717/peerj.2587

**Published:** 2016-11-10

**Authors:** Catherine Gérikas Ribeiro, Adriana Lopes dos Santos, Dominique Marie, Vivian Helena Pellizari, Frederico Pereira Brandini, Daniel Vaulot

**Affiliations:** 1 Departamento de Oceanografia Biológica, Instituto Oceanográfico, Universidade de São Paulo, São Paulo, Brazil; 2 Sorbonne Universités, UPMC Université Paris 06, CNRS, UMR 7144, Station Biologique de Roscoff, France

**Keywords:** Picoplankton, Nanoplankton, *Prochlorococcus*, *Synechococcus*, Heterotrophic bacteria, Flow cytometry, Southwest Atlantic Ocean off Brazil

## Abstract

Pico and nanoplankton communities from the Southwest Atlantic Ocean along the Brazilian Bight are poorly described. The hydrography in this region is dominated by a complex system of layered water masses, which includes the warm and oligotrophic Tropical Water (TW), the cold and nutrient rich South Atlantic Central Water (SACW) and the Coastal Water (CW), which have highly variable properties. In order to assess how pico- and nanoplankton communities are distributed in these different water masses, we determined by flow cytometry the abundance of heterotrophic bacteria, *Prochlorococcus*, *Synechococcus* and autotrophic pico and nanoeukaryotes along three transects, extending from 23°S to 31°S and 39°W to 49°W. Heterotrophic bacteria (including archaea, maximum of 1.5 × 10^6^ cells mL^−1^) were most abundant in Coastal and Tropical Water whereas *Prochlorococcus* was most abundant in open-ocean oligotrophic waters (maximum of 300 × 10^3^ cells mL^−1^). *Synechococcus*(up to 81 × 10^3^ cells mL^−1^), as well as autotrophic pico and nanoeukaryotes seemed to benefit from the influx of nutrient-rich waters near the continental slope. Autotrophic pico and nanoeukaryotes were also abundant in deep chlorophyll maximum (DCM) layers from offshore waters, and their highest abundances were 20 × 10^3^ cells mL^−1^ and 5 × 10^3^ cells mL^−1^, respectively. These data are consistent with previous observations in other marine areas where *Synechococcus* and autotrophic eukaryotes dominate mesotrophic waters, whereas *Prochlorococcus* dominate in more oligotrophic areas. Regardless of the microbial community structure near the surface, the carbon stock dominance by autotrophic picoeukaryotes near the DCM is possibly linked to vertical mixing of oligotrophic surface waters with the nutrient-rich SACW and their tolerance to lower light levels.

## Introduction

The microbial communities of the Southwest Atlantic Ocean (SAO) off Brazil are just beginning to be investigated (Buitenhuis et al., 2012; Alves Junior et al., 2015). A complex system of layered water masses structures the primary productivity along the SAO near the Brazilian Bight. The South Atlantic Central Water (SACW) has an oceanic origin and is situated below the Tropical Water (TW), being represented in the Temperature/Salinity (T–S) diagram as a straight line between T-5 °C/S-34.3 and T-20 °C/S-36 (Sverdrup, Johnson & Fleming, 1942; Stramma & England, 1999). The SAO western boundary system (below 20°S) is mainly influenced by a wind-driven system and the seasonal (spring-summer) intrusion of the nutrient-rich SACW along the bottom of the continental shelf (Campos, Velhote & Da Silveira, 2000; Castro et al., 2006). SACW can also be pumped by cyclonic meanders of the Brazil Current, which consists of rotating domes of upwelled, cold water that flows inshore through the shelf break (Campos, Velhote & Da Silveira, 2000). The Brazil Current is shallow (*ca* 200 m), restricted to the shelf break and flows southwestward towards the Brazil-Malvinas Confluence Zone (Brandini et al., 2000) transporting the warm (T > 20 °C), saline (S > 36) and nutrient-poor TW (Emílsson, 1961). The Coastal Water (CW) originates through characteristic processes of the inner portions of continental shelves, such as fresh water discharges and estuarine plumes, and its main features are low salinity (S < 35) and high spatial and seasonal variability (Castro et al., 2006).

Although the oligotrophic TW dominates the SAO euphotic zone, its rate of primary production is higher than in subtropical gyres (Brandini, 1990a). Diatoms, dinoflagellates, coccolithophorids and cyanobacteria are amongst the most abundant groups of planktonic primary producers in this region (Brandini, 1990b; Fernandes & Brandini, 2004; Susini-Ribeiro & Pompeu, 2013; Brandini et al., 2014; Moser et al., 2014). A few studies have examined the influence of different water masses on micro-phytoplankton composition (Brandini et al., 2014; Moser et al., 2014) and primary production (Brandini, 1990a; Brandini, 1990b) in this region. The uplift of the SACW promotes euphotic layer fertilization (Brandini, 1990b) through a shift from regenerated to new production (Metzler et al., 1997) influencing the structure of micro-phytoplankton communities (Susini-Ribeiro & Pompeu, 2013; Brandini et al., 2014; Moser et al., 2014). However, the influence of such processes on the smaller size classes of the phytoplankton remains to be clarified. Although previous studies suggest a high importance of picoplankton, which may account for up to 64% of the total carbon biomass (Susini-Ribeiro, 1999), little is known about the pico-phytoplankton abundance, diversity and response to the hydrodynamic regime in the SAO off Brazil.

Pico- and nano-phytoplankton, defined as cells within the size range of 0.2–2 and 2–20 µm, respectively, include both photosynthetic prokaryotes and eukaryotes, and its significance arises from its ubiquity, abundance and persistency in aquatic environments. These size classes have a strong impact on the primary production and carbon cycling in the marine environment (Li, 1994; Worden et al., 2004; Grob et al., 2007; Richardson & Jackson, 2007). Despite its small size compared to the other components of the plankton, pico-phytoplankton cells are important carbon export agents, via either aggregate formation or consumption by higher trophic level organisms (Richardson & Jackson, 2007). Pico-phytoplankton may account to up to 60% of the chlorophyll-*a* and primary production in some regions of the Atlantic Ocean (Pérez et al., 2005), with greatest contribution in tropical and oligotrophic waters (Agawin, Duarte & Agusti, 2000).

Within pico-phytoplankton, the cyanobacterium *Prochlorococcus* is widespread in the euphotic zone of the tropical and subtropical oceans (De Corte et al., 2016), and is considered the smallest and most abundant photosynthetic organism on the planet (Partensky, Hess & Vaulot, 1999). Its broad genomic and phenotypic diversity are probably key factors explaining its wide distribution (40°N–40°S) (Johnson, 2006) and its high and stable abundance throughout the oceans (Kashtan et al., 2014; Biller et al., 2014). It is considered to be responsible for a projected carbon fixation of 4 Gt C y^−1^, or approximately 9% of ocean’s net primary production (Flombaum et al., 2013). *Synechococcus*, the other important picoplanktonic cyanobacterium genus present, is highly diverse with more than 20 genetically distinct clades (Sohm et al., 2015) widely distributed in marine ecosystems (Zubkov et al., 1998; Partensky, Blanchot & Vaulot, 1999), from cold and mesotrophic to warm open ocean oligotrophic waters. *Synechococcus* may account for up to 17% of net primary production in the oceans (Flombaum et al., 2013) and it has been recently associated with high carbon export rates in subtropical, nutrient depleted waters (Guidi et al., 2016). Photosynthetic pico and nanoeukaryotes display a range of physiologies and life strategies, with Chlorophyta, Heterokontophyta, and Haptophyta being the most important groups (Worden & Not, 2008). Although less abundant than *Synechococcus* and *Prochlorococcus*, through equivalent growth and larger cell size, picoeukaryotes can dominate carbon production and biomass in oceanic and coastal waters (Zubkov et al., 1998; Worden et al., 2004; Worden & Not, 2008; Guo et al., 2014).

The goal of the present study is to describe the spatial distribution of heterotrophic bacteria as well as pico and nano-phytoplankton in cross-shelf transects along the Brazilian Bight in order to assess their population structure and contribution to carbon standing stocks in the different water masses.

## Materials and Methods

### Sampling

**Figure 1:**
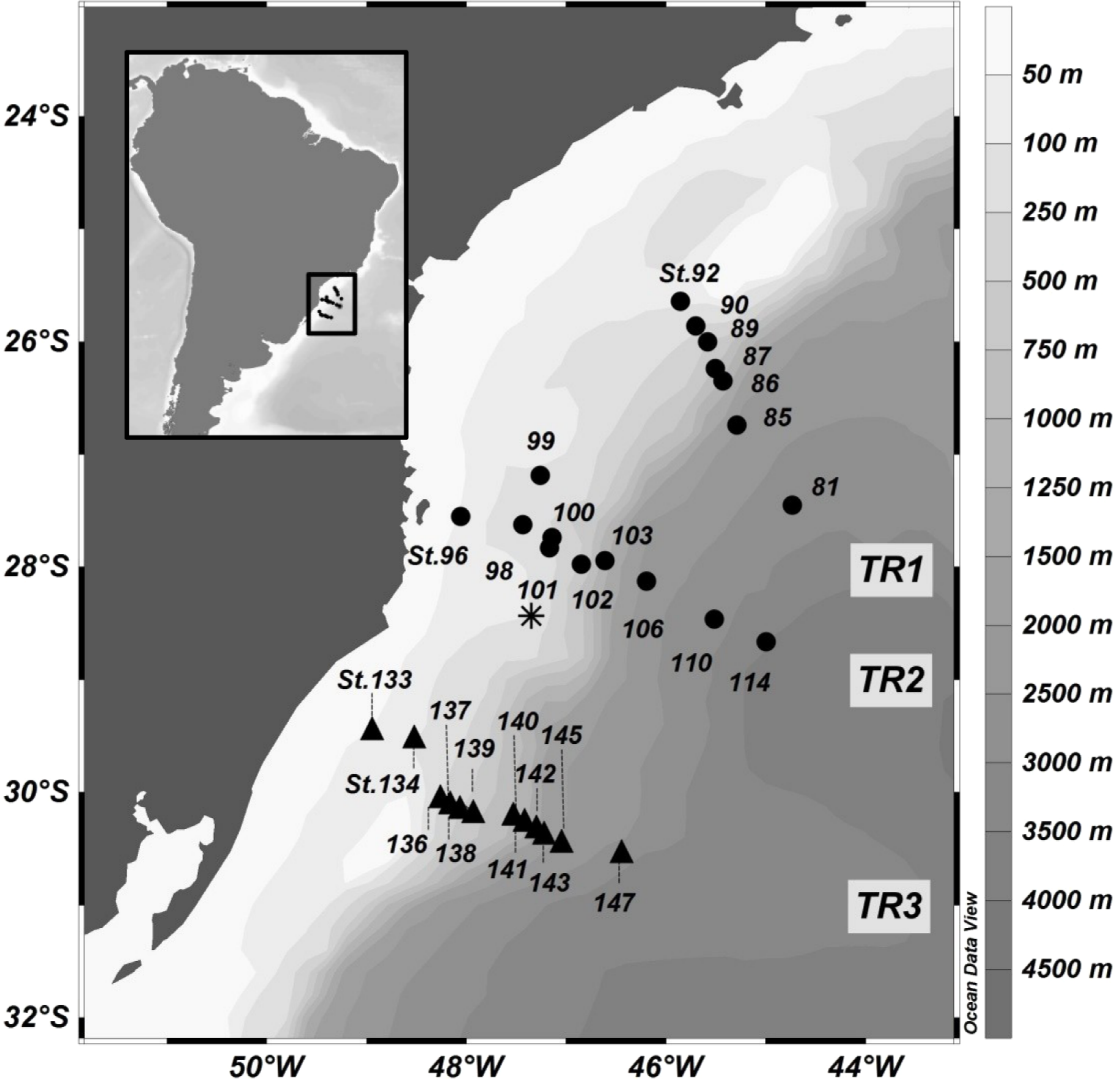
Location of sampling stations in the SAO off Brazil (11–18 November 2013). Profiles: transect 1 (TR1, St.81 to 92) and transect 2 (TR2, St.96 to 114), represented by black dots; Surface sampling: transect 3 (TR3, St.133 to 147), represented by black triangles. The asterisk represents the TRICHO station, located inside a *Trichodesmium* sp. bloom.

Seawater samples were collected in the Southwest Atlantic off Brazil onboard the R/V “Alpha Crucis”, between October and November 2013. The surveyed area is located between latitudes 23°11′S–30°52′S and longitudes 39°22″W–49°09″W, extending to the 3,510 m isobath, along 2 transects (TR1 and TR2), comprising five depths per profile, and a third auxiliary transect called TR3, with only surface samples (Fig. 1). A *Trichodesmium* sp. bloom was observed during TR2, for which additional sampling was performed at the surface (Station TRICHO). All samples were collected in a rosette system with 12 L Niskin bottles attached to a CTD Teledyne model PS7000M (Teledyne Technologies Inc, CA, USA), except for surface samples from TR3 and TRICHO, which were collected with a polycarbonate bucket. The temperature and salinity data from CTD were used to identify the distribution of the water masses during the transects. Duplicate samples (1.5 mL) for flow cytometry (FCM) were collected into cryotubes, preserved with 0.1% glutaraldehyde (final concentration), flash-frozen in liquid nitrogen and stored at −80 °C until analysis.

### Nutrient analysis

For nitrate and phosphate analysis, samples were filtered through Whatman^®^ GF/F filters using a vacuum pump. The filtered water was frozen at −20 °C until laboratory analysis by the colorimetric methods described in Hansen & Koroleff (1999) using a spectrophotometer Hitachi^®^ U-1000.

### Flow cytometry analysis

Flow cytometry analysis was performed as previously described in Marie et al. (2000) and Ribeiro et al. (2016) using a BD FACSCanto II™(Becton Dickinson, San Jose, CA) flow cytometer equipped with a blue laser (488 nm, air-cooled, 20 mW, solid state). Emitted light was collected through the following set of filters: 488/10 band pass for side scatter, 533/30 band pass for green SYBR fluorescence (FL1), 585/42 band pass for orange phycoerythrin fluorescence (FL2), and 670 long pass for red chlorophyll fluorescence (FL3). Samples were thawed at room temperature, and 0.95 µm beads (0.95 G Fluoresbrite^®^ Polysciences, Warrington, PA, USA) were used for FCM calibration. A first analysis of 3 min at a rate of 70 µL min^−1^ was performed to enumerate phytoplankton cells. Acquisition was triggered on chlorophyll fluorescence (FL3-H ), which therefore excluded any heterotrophic cell, using a threshold of 200. A second analysis was performed in order to enumerate heterotrophic prokaryotes: SYBR Green^®^ (Molecular Probes, Leiden, Netherlands) was added at a final concentration of 1/10,000 and samples were incubated for at least 15 min at room temperature in the dark. Flow cytometry acquisition was triggered on FL1 with a threshold value of 500 and performed for 2 min with a flow rate of 60 µL min^−1^. Data were analyzed with the Flowing Software^®^ 2.5 (http://www.flowingsoftware.com). For phytoplankton, chlorophyll and phycoerythrin fluorescence, as well as forward and side scatter were used to distinguish between four major groups: *Prochlorococcus*, *Synechococcus*, pico-phytoeukaryotes and nano-phytoeukaryotes (Fig. S1). For SYBR Green^®^ stained samples, only prokaryotes (called throughout the paper heterotrophic bacteria, including possibly Archaea) were included in the analysis to the exclusion of any heterotrophic eukaryotes.

Picoplankton biomass (heterotrophic bacteria, *Prochlorococcus*, *Synechococcus* and picoeukaryotes) was calculated from flow cytometry abundance data, using cell-to-carbon conversion factors from the literature: 20 fgC cell^−1^ for heterotrophic bacteria (Lee & Fuhrman, 1987), 36 fgC cell^−1^ for *Prochlorococcus*, 255 fgC cell^−1^ for *Synechococcus*, and 2,590 fgC cell^−1^ for picoeukaryotes (Buitenhuis et al., 2012). Nano-phytoplankton biomass was not calculated due the lack of robust conversion factors to carbon content.

### Data analysis

Statistical analyses were made with the STATISTICA 12.5^®^ software (Version 13; StatSoft, Inc., Tulsa, OK, USA) in order to explore relationships between abiotic and biotic data. A Spearman correlation analysis was performed considering both environmental (temperature, fluorescence, salinity, phosphates and nitrates) and biotic data (heterotrophic bacteria, *Prochlorococcus*, *Synechococcus*, picoeukaryote and nanoeukaryote abundances). A Principal Component Analysis (PCA, *N* = 72) was performed with abiotic and biotic data computed as active and supplementary variables, respectively. Graphic interpolations were produced with the *DIVA Gridding* algorithm from the software Ocean Data View^®^ version 4.7.6 (Schlitzer, 2016). Flow cytometry and environmental data (Table S1) can be found at https://dx.doi.org/10.6084/m9.figshare.3492098.v2.

## Results

### Environmental conditions

**Figure 2:**
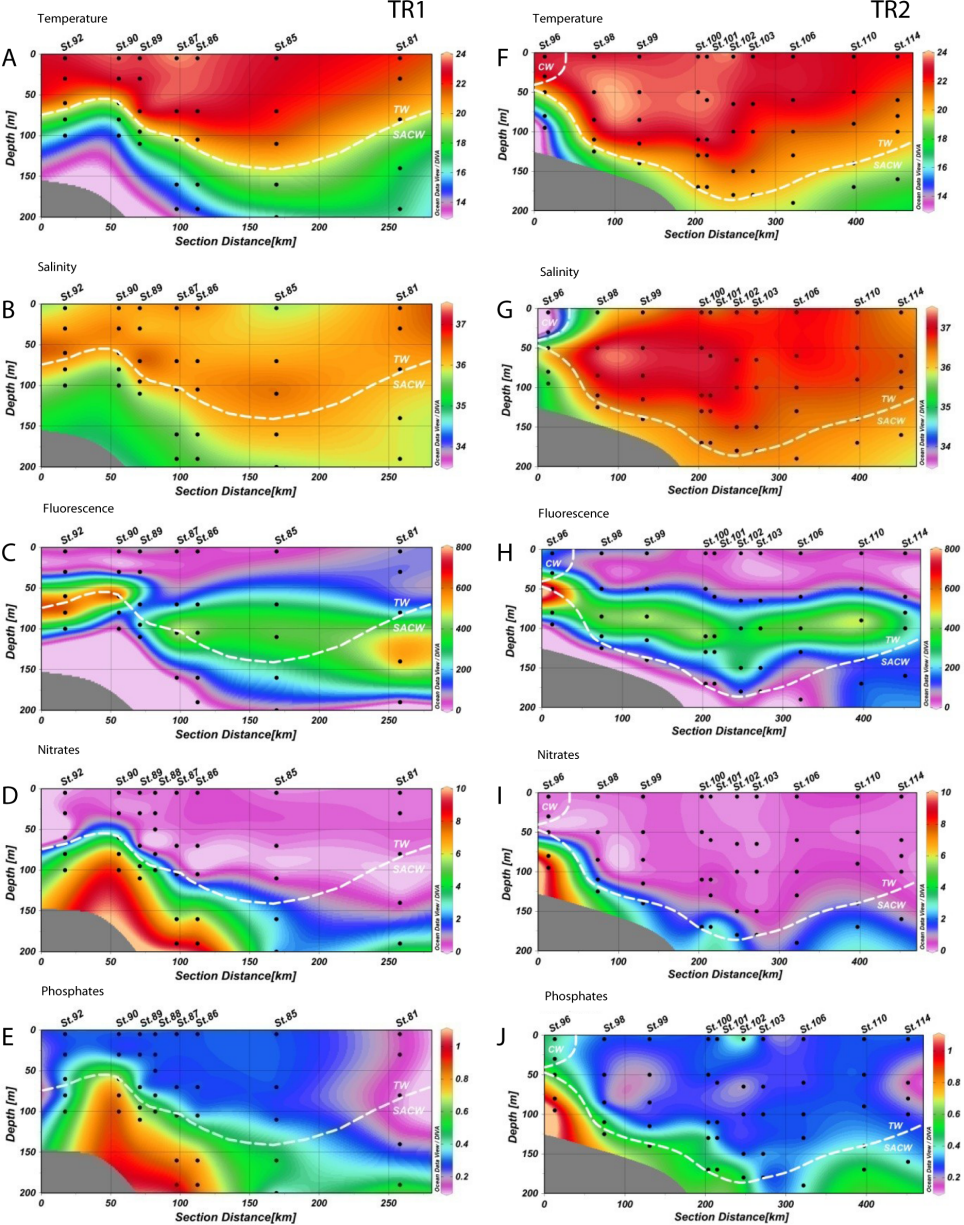
Distribution of environmental variables. Vertical distributions (from the top) of temperature (T °C), salinity, fluorescence (RFU), nitrates (µM) and phosphates (µM) for transect 1 (TR1, A–E) and transect 2 (TR2, F–J); numbers indicate sampling stations; dashed white lines represent the boundary between water masses; TW, Tropical Water; SACW, South Atlantic Central Water; CW, Coastal Water.

Three main SAO pelagic water masses were sampled in this study: Coastal Water (TR2, TR3), Tropical Water (TR1, TR2, TR3) and South Atlantic Central Water (TR1, TR2). A rise of the thermocline, as well as a minor SACW elevation in the outermost stations were observed in both TR1 and TR2 (Fig. 2).

Temperature varied from 12.8 °C to 23.8 °C in TR1 and from 13.9 °C to 23.8 °C in TR2. Salinity variation was considerably narrower, ranging from 35.1 to 36.8 on TR1 and from 33.4 to 37.2 in TR2. Tropical Water along the TR2 surface mixed layer was more saline and slightly warmer than TR1 (Figs. 2A, 2B, 2F and 2G). For the surface transect TR3, temperature ranged from 20.2 °C to 23 °C, whilst a wider salinity range (from 33.5 to 36.8) was observed, indicating the presence of coastal waters at the inner stations (Figs. S2A and S2B).

The rise of the thermocline over the continental slope induced both upward displacement and enhancement of the DCM (Figs. 2C and 2H). Chlorophyll fluorescence ranged from 2.7 to 780 RFU (Relative Fluorescence Units), with median values of 191 and 187 RFU in TR1 and TR2, respectively. A DCM was present at all stations along TR1 and TR2, with an enhancement and upward displacement by up to 50 m in TR1 due to thermocline rise (Figs. S2C and S2H). In TR3, the fluorescence ranged from 17 to 210 RFU (Table S1).

The nutricline was sharply defined in TR1 and TR2, more or less coincident with the SACW upper limit (Figs. 2D, 2E, 2I and 2J). Local concentration maxima were observed near the bottom of the continental shelf (8.7 and 9.1 µM of nitrates, 1 and 1.1 µM of phosphates for TR1 and TR2, respectively). CW was characterized by low nutrient concentration, with the exception of a slightly increase in phosphates (up to 0.4 µM) while nitrates were close to depletion in all surface samples (Figs. S2D and S2E).

### Abundance of microbe populations

**Figure 3:**
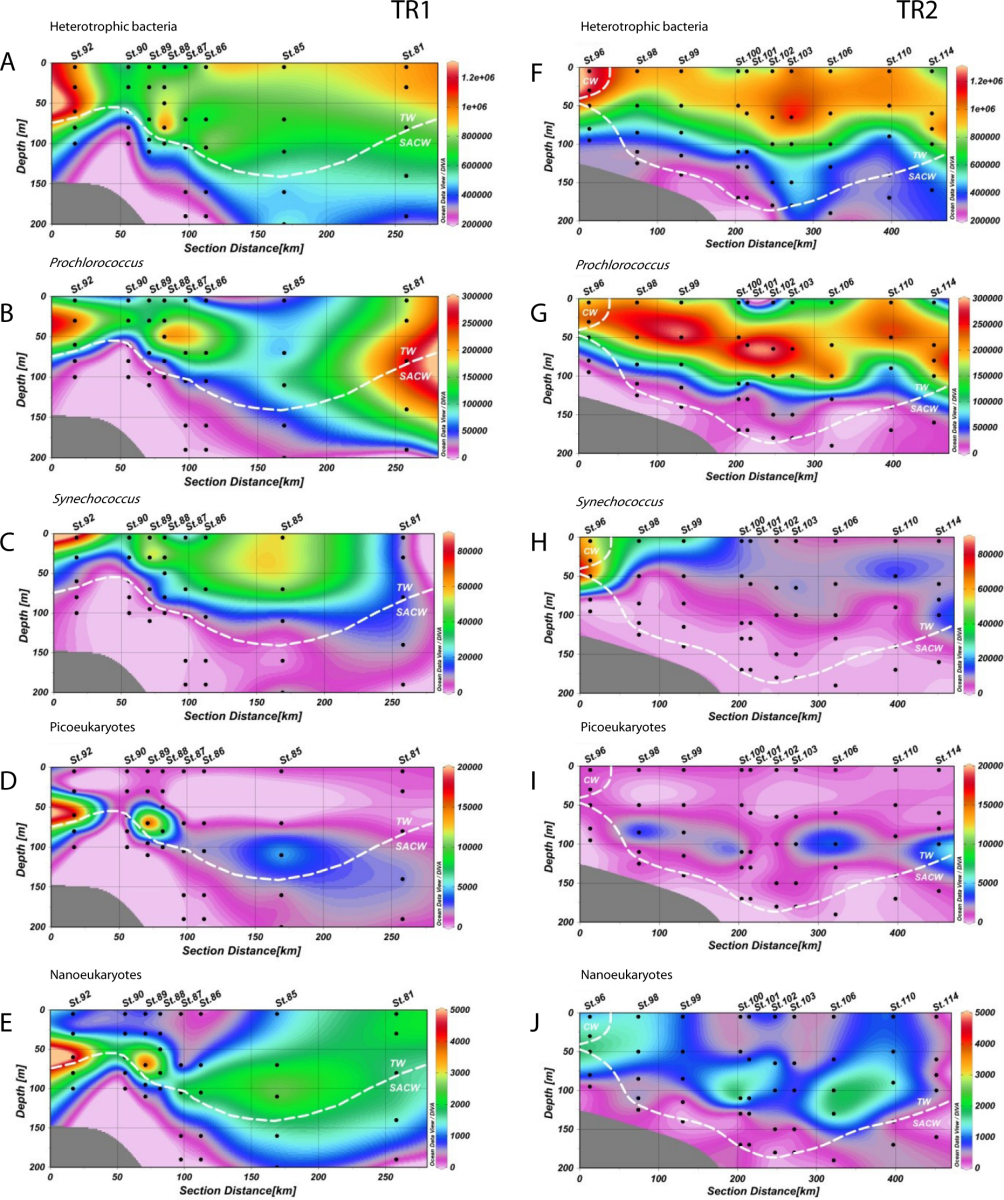
Distribution of biological variables. Vertical abundance distributions (from the top) of total heterotrophic bacteria, *Prochlorococcus*, *Synechococcus*, picoeukaryotes and nanoeukaryotes (in cells.mL^−1^) for transect 1 (TR1, A–E) and transect 2 (TR2, F–J); numbers indicate sampling stations; dashed white lines represent the boundary between water masses; TW, Tropical Water; SACW, South Atlantic Central Water; CW, Coastal Water.

Heterotrophic bacteria populations were abundant near the shelf break in both transects and throughout TW in TR2 (Figs. 3A and 3F). The rise of the thermocline induced an increase in the abundance of this group in both transects, with maxima of 1.3 × 10^6^ and 1.2 × 10^6^ cells mL^−1^ in TR1 and TR2, respectively. The innermost station of TR3 had the highest abundance of heterotrophic bacteria of the cruise (1.5 × 10^6^ cells mL^−1^, Fig. S3A). Abundance maxima were found from the surface layer down to 80 m in TR1 (1.1 × 10^6^ cells mL^−1^) and 65 m in TR2 (1.2 × 10^6^ cells mL^−1^). The presence of a *Trichodesmium* sp. bloom in TR2 caused a sharp increase in heterotrophic bacteria abundance with 3 × 10^6^ cells mL^−1^ (Table S1).

*Prochlorococcus* abundances were higher in CW and TW surface waters (Figs. 3B and 3G). In TR1, higher *Prochlorococcus* abundances were coincident with upward movements of SACW, reaching the lower limit of the DCM (Fig. 3B), with high abundance maxima at the inner and outermost stations (230 × 10^3^ and 261 × 10^3^ cells mL^−1^, respectively). *Prochlorococcus* abundance was higher and its distribution was more stratified in TR2. These higher concentrations (290 × 10^3^ cells mL^−1^) were located in the subsurface layers. In the innermost station of TR2, the upper limit of the thermocline and the presence of CW did not seem to influence *Prochlorococcus* concentrations. The highest abundance (266 × 10^3^ cells mL^−1^) of *Prochlorococcus* in TR3 occurred in the middle of the transect, distal from the SACW influence. *Prochlorococcus* were virtually absent at the TRICHO station (Table S1).

The highest abundances of *Synechococcus* were found in the surface layer. Concentrations were high throughout the TW in TR1 (Fig. 3C). Maximum abundance (81 × 10^3^ cells mL^−1^) was found in the surface at St.92, which was the sampled station closest to the coast. In TR2, the highest concentration of *Synechococcus* (67 × 10^3^ cells mL^−1^) was also found near the shelf break, at 50 m depth. In contrast to TR1, TR2 *Synechococcus* distribution was more confined to the continental shelf (Fig. 3H), especially in CW, and in the transition waters from CW to TW. In TR3, only sampled at the surface, *Synechococcus* maximum was also observed at the innermost station, with 39 × 10^3^ cells mL^−1^ (Fig. S3C). The maximum *Synechococcus* abundance from the cruise, 335 × 10^3^ cells mL^−1^, was observed at TRICHO station (Table S1).

Photosynthetic picoeukaryote populations in TR1 increased in conjunction with the rise of the thermocline, as can be observed at St.92 (Fig. 3D, 18 × 10^3^ cells mL^−1^). Two local abundance maxima were observed along the transect (18 × 10^3^ and 4 × 10^3^ cells mL^−1^), deepening along with SACW and coinciding with the DCM. Lower abundance values were found in TR2 without any increase at inner stations. TR2 abundance maxima (3 × 10^3^, 4 × 10^3^ and 5 × 10^3^ cells mL^−1^) were distributed along the transect, near the DCM (Fig. 3I). Picoeukaryote abundances were low in surface for all transects including TR3, for which maximum abundance was 2 × 10^3^ cells mL^−1^ at the innermost station (Fig. S3D).

Photosynthetic nanoeukaryote distribution along TR1 was very similar to picoeukaryote distribution at the inner stations of the transect, but beyond the shelf break nanoeukaryote distribution extended more vertically, reaching both the surface layer and the lower limit of the DCM. Maximum abundance was right above the thermocline (5 × 10^3^ cells mL^−1^, Fig. 3E). In common with picoeukaryotes, nanoeukaryote abundance in TR2 appears to have a close relationship with the DCM along the transect, although an increase up to 1.6 × 10^3^ cells mL^−1^ could be observed at the CW/SACW intersection (St. 96, at 50 m depth, Fig. 3J). In TR3, nanoeukaryote surface distribution was very patchy and ranged from 0.2 × 10^3^ to 1.3 × 10^3^ cells mL^−1^ (Fig. S3E).

According to Spearman’s rank correlation coefficients all populations were negatively correlated with nutrients, except for picoeukaryotes, and positively correlated with temperature (also except for picoeukaryotes). Pico and nanoeukaryotes were significantly correlated with fluorescence, whilst only *Prochlorococcus* was influenced by salinity (Table 1).

**Table 1:** Spearman’s rank correlation coefficient between environmental factors and abundances of picophytoplankton populations.

	T(°C)	Fluo	Sal	NO^3−^	PO4^3−^
Heterotrophic bacteria	0.55^*^	−0.05	−0.07	−0.74^*^	−0.47^*^
*Prochlorococcus*	0.61^*^	0.16	0.26^*^	−0.72^*^	−0.67^*^
*Synechococcus*	0.60^*^	−0.01	−0.15	−0.76^*^	−0.43^*^
picoeukaryotes	0.07	0.47^*^	0.01	−0.22^*^	−0.15
nanoeukaryotes	0.23^*^	0.46^*^	0.00	−0.39^*^	−0.24^*^

**Notes.**

* Correlation significant at 0.05 level; *n* = 102.

**Figure 4:**
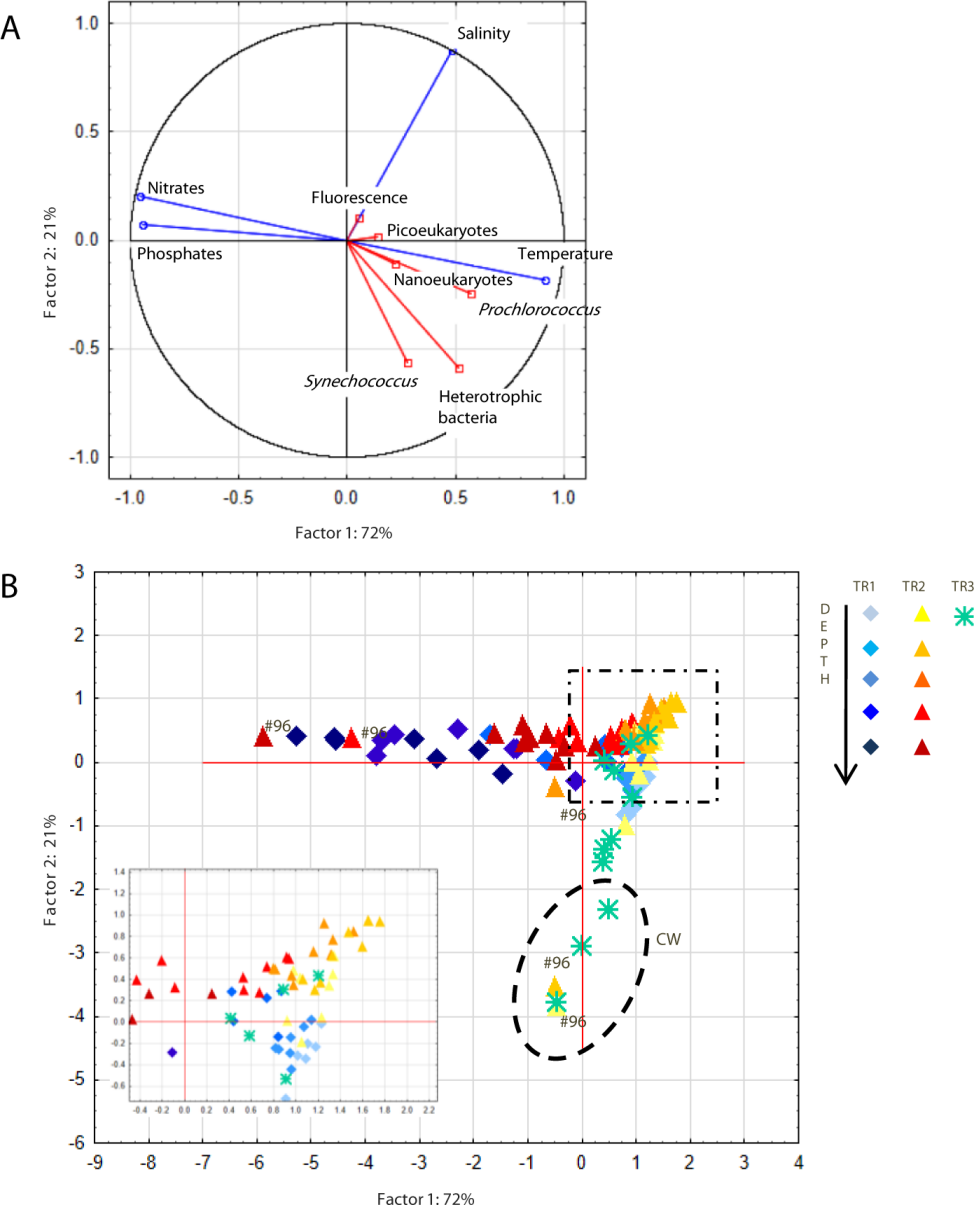
Principal component analysis. Principal component analysis (PCA) showing (A) PC1 and PC2 plot of environmental (temperature, salinity, fluorescence, nitrates and phosphates) and biological (heterotrophic bacteria, *Prochlorococcus*, *Synechococcus*, picoeukaryotes and nanoeukaryotes) variables. Abiotic and biotic data were computed as active (blue) and supplementary (red) variables, respectively. (B) Station scores of PC1 and PC2. Samples are indicated by diamonds (TR1), triangles (TR2) and asterisks (TR3). Dashed ellipse indicates samples from Coastal Water (CW), dashed square indicates the zoom window; #96 refers to samples from St. 96.

The first two components of a PCA based on temperature, salinity, nitrates and phosphates explained 93% of the observed variability (Fig. 4A). The first axis correlated positively with temperature and negatively with nutrients, which reflects the influence of cold nutrient-rich SACW, while the second axis correlated positively with salinity. *Prochlorococcus*, nanoeukaryotes and picoeukaryotes correlated with the first axis while *Synechococcus* and heterotrophic bacteria appeared to be influenced by both axes. Due to the influence of TW higher salinity, samples from TR2 were more clustered together (top right quadrant, Fig. 4B) than for TR1. The innermost station of TR2 (St.96) located at the intersection of CW and SACW (Fig. 2) displayed very scattered data points (Fig. 4).

### Carbon biomass of picoplankton populations

**Figure 5:**
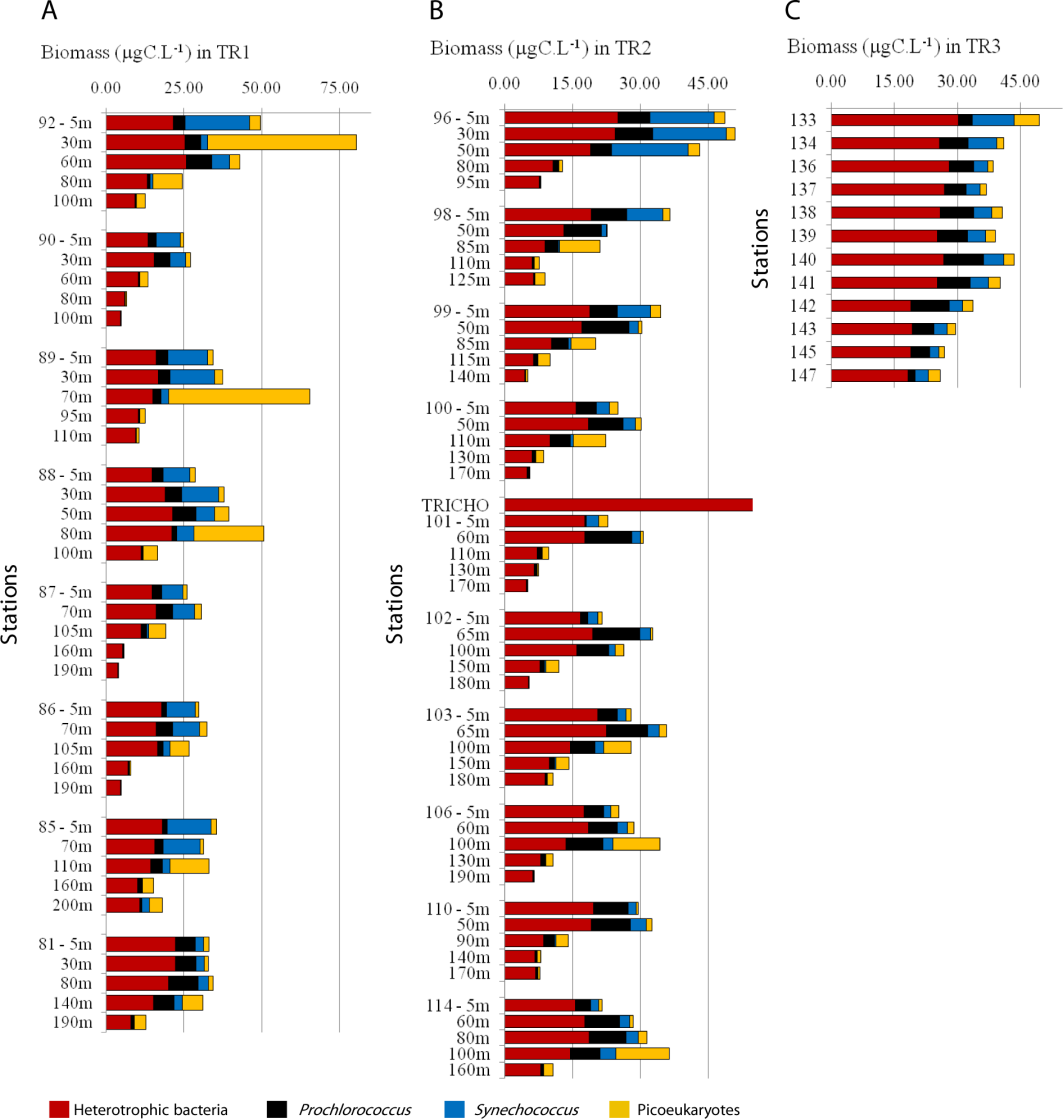
Biomass (µgC mL^−1^) estimated for total heterotrophic bacteria (in red), *Prochlorococcus* (black), *Synechococcus* (blue) and picoeukaryotes (yellow) for TR1, TR2 and TR3. Note that the scale is different for TR1 compared to TR2 and TR3.

Heterotrophic bacteria biomass ranged from 4 to 33 µgC L^−1^, and dominated carbon picoplankton biomass (67% on average, ranging from 26 to 99%) (Fig. 5; Figs. S4B and S4G). *Prochlorococcus* contributed more to total pico-phytoplanktonic biomass in oligotrophic and warmer TW, reaching 66% (9 µgC L^−1^, TR1, St. 81, 80 m depth) and 87% (8 µgC L^−1^, TR2, St. 98, 50 m depth) of total autotrophic carbon (Fig. 5; Fig. S4C and S4H). *Prochlorococcus* mean relative contribution to total autotrophic biomass was 22%, 43% and 48% in TR1, TR2 and TR3, respectively. *Synechococcus* biomass contribution to total autotrophic biomass was high throughout TW in TR1 (81%, St. 85) and at the innermost stations, mainly in CW and shelf waters, in TR2 (70%, St. 96) and TR3 (maximum of 53%, St. 133) (Fig. 5; Fig. S4D and Fig. S4I). The relative importance of picoeukaryote biomass was higher in deeper samples near DCM, reaching 90% of total autotrophic biomass in TR1 (St. 89) and 91% in TR2 (St. 98). On average, picoeukaryotes contributed to 25% of the pico-phytoplankton biomass, falling below 15% in the uppermost layers of TW (Fig. 5; Figs. S4E and S4J).

## Discussion

One of the most important processes for primary productivity in the oligotrophic offshore waters of the SAO is the uplifting of the nutrient-rich South Atlantic Central Water and its interactions with Tropical Water and Coastal Water (Castro et al., 2006), leading to spatial variations in temperature, salinity, nutrients and light availability (Brandini, 1990a; Brandini et al., 2014; Moser et al., 2014). Salinity and temperature profiles from TW change as it flows southwards, as a consequence of the loss of heat by evaporation (Emílsson, 1961). Due to this increase in salinity and density in TR2, the TW reached deeper layers in the water column. Apart from some phosphate enrichment, low nutrient concentrations were found in CW (Figs. 2D, 2E, 2I and 2J, Figs. S2D and S2E), which is expected, since the region is not influenced by any significant continental drainage. Vertical chlorophyll fluorescence maxima seem to be correlated with the thermocline elevation near the continental slope, and a prominent DCM layer is visible throughout the transects TR1 and TR2 (Figs. 2C and 2H). SACW upward displacement in the outermost stations could be attributed to either meandering activity or internal gravity waves, which are known to be able to raise the thermocline into the euphotic zone (Johannessen, 1968), and may be responsible for the increase in fluorescence at depth at St. 81 in TR1.

### Contribution of the different microbe populations

Similar ranges of the abundance of each planktonic group were reported for other coastal shelf systems and oligotrophic oceanic waters (Zubkov et al., 1998; Katano et al., 2005). In general, heterotrophic bacteria distribution tends to follow pico-phytoplankton biomass structure along the water column, as reported before in the Atlantic Ocean (Zubkov et al., 1998; Zubkov et al., 2000). Abundance values for heterotrophic bacteria reported here (0.2 × 10^6^ − 1.5 × 10^6^ cells mL^−1^) are consistent with those found in other studies in different regions along the Brazilian coast (Andrade et al., 2003; Andrade & Gonzalez, 2007), in the Atlantic Ocean (Zubkov et al., 1998), and in other marine ecosystems (Grob et al., 2007; Herfort et al., 2012; Šilović et al., 2012). The increased abundance of heterotrophic bacteria near the thermocline rise (Figs. 3A and 3F) could be related to the accumulation of dissolved and particulate organic matter in this frontal region (Linacre et al., 2015).

*Prochlorococcus* contribution to primary productivity in oligotrophic regions is well documented (Biller et al., 2014), and its abundance tends to peak in highly stratified upper layers (Johnson, 2006), with a wide vertical distribution linked to the coexistence of differently adapted ecotypes (West et al., 2001; Bouman, 2006; Farrant et al., 2016). *Prochlorococcus* outnumbered the other pico-phytoplanktonic groups in all transects, with a mean concentration of 100 × 10^3^ cells mL^−1^. The observed concentration range agrees with reports on the western boundary of the South Atlantic Gyre (Zubkov et al., 2000; Flombaum et al., 2013; De Corte et al., 2016) as well as in other marine ecosystems (Partensky, Hess & Vaulot, 1999). *Prochlorococcus* local maxima appear to be correlated with the upper edge of the thermocline rise in TR1. In TR2, *Prochlorococcus* was particularly abundant over the first 100 m of the oligotrophic TW. The significant negative correlation between *Prochlorococcus* and nitrates is expected (Table 1, Fig. 4), since most *Prochlorococcus* strains lack the genes required for NO_3_ uptake and reduction (Moore et al., 2002).

*Synechococcus* high cell abundances are associated with the presence of tropical and sub-tropical mesotrophic waters and upwelling events (Zubkov et al., 1998; Van Dongen-Vogels et al., 2011). In the present study, *Synechococcus* higher abundances (up to 81 × 10^3^ cells mL^−1^) were found mostly in superficial, shelf waters, in association with the thermocline upward movement (Figs. 5C and 5H). The highest abundances found on the three transects suggest coastward enhancement on *Synechococcus* populations, which has been reported in other studies (Jiao et al., 2002), but also a decrease towards the South (Fig. S3C). Latitudinal abundance shifts are expected, since *Synechococcus* niche partitioning can be dictated by individual clade preferences for temperature, macronutrients and iron availability (Sohm et al., 2015). Inside the *Trichodesmium* sp. bloom observed in this study (St. TRICHO) there was an approximately 30-fold increase in *Synechococcus* abundance (Table S1), compared to the nearest superficial station (St. 101). A similar pattern (a 10-fold increase in *Synechococcus* abundances) was reported previously inside a *Trichodesmium* sp. bloom, in the Southwest Pacific (Campbell et al., 2005). The presence of a superficial bloom does not appear to influence populations in deeper layers of the water column, even though *Trichodesmium* sp. is known to export to deeper waters up to 90% of its recently fixed nitrogen (Mulholland & Bernhardt, 2005).

The mean picoeukaryotic abundance obtained in this study (1.34 × 10^3^ cells mL^−1^) is in accordance with averages observed in oligotrophic waters (Zubkov et al., 1998; Worden & Not, 2008). Beyond the shelf break, picoeukaryote abundances peaked in deeper samples, between 50 m and 100 m water depth (Figs. 3D and 3I). Picoeukaryote populations often form a maximum in deeper layers, in tropical and subtropical oligotrophic waters, and an upper layer maximum, when upwelling or frontal systems pump nutrient rich waters into the euphotic zone (Zubkov et al., 1998; Jiao et al., 2002). The close relationship between DCM layers and picoeukaryote abundance reported here has been previously described at the western boundary of the southern Atlantic Gyre (Zubkov et al., 2000) and close to its center (Tarran, Heywood & and, 2006). Painter et al. (2014) observed maximum picoeukaryote abundance coinciding with maximum NO_3_ uptake rates, near the nitracline, which indicate that these populations may be major players for production in the deeper layers of the euphotic zone, fueling the downward flux of carbon to the ocean interior through the formation of aggregates (Lomas & Moran, 2011). This dominance at the DCM could be linked to their better adaptation to low light levels compared to larger phytoplankton and *Synechococcus* (Worden & Not, 2008).

Nanoeukaryote higher abundances were distributed through a wider depth range than picoeukaryotes, extending from the surface down to 200 m depth at some stations (Fig. 3E), which may reflect distinct light and nutrient preferences of a more diverse assemblage of taxa (Marie et al., 2010).

### Influence of water masses on microbial population

Pico-phytoplankton dominates biomass in nutrient poor, warm waters (Agawin, Duarte & Agusti, 2000), and the same is observed for the oligotrophic waters of the SAO (Marañón et al., 2003). In TR2 and TR3, the presence of Coastal Water increased autotrophic carbon standing stocks. The mean autotrophic picoplankton carbon concentration measured in this study (21 µgC L^−1^) is similar to global estimates for tropical regions (Buitenhuis et al., 2012), and accounted, on average, for 38% of the total microbial biomass (including heterotrophic bacteria). The biomass distribution was particularly homogeneous in TR1 beyond the shelf break (Fig. S4A), despite the sharp enhancement in total pico-phytoplankton biomass near the thermocline raise. The percentage of autotrophic biomass was strongly linked to the upward displacement of the thermocline/nutricline over the shelf break, reaching up to 77% of total biomass (Fig. S4A). This is comparable to the highest measurements observed in the Atlantic Ocean (Marañón et al., 2001; Pérez et al., 2005).

The physical structure of the oligotrophic waters near continental shelves has a significant impact on the relative dominance of each picoplankton group (Jiao et al., 2002; Van Dongen-Vogels et al., 2011; Linacre et al., 2015). In the present study, heterotrophic bacteria accounted for a large fraction of picoplankton carbon in deeper samples where autotrophic populations decreased, whilst *Synechococcus* and picoeukaryote biomass were more important near the thermocline. *Synechococcus* and picoeukaryote biomass enhancements have been linked to the destabilization of the water column caused by upwelling process in other coastal shelf systems (Van Dongen-Vogels et al., 2011). The prominent dominance of *Synechococcus* biomass throughout TW in TR1 may indicate a higher (picoplankton driven) carbon export to deeper layers than in TR2, since the presence of this group have been linked to an increase in the efficiency of the biological carbon pump (Guidi et al., 2016). Although outnumbered by most of other picoplankton groups, the biomass from picoeukaryotes comprised a substantial fraction of the autotrophic carbon, particularly in deeper samples, which has been previously observed in oligotrophic Atlantic waters (Zubkov et al., 1998). The variation in dominance patterns between the transects, switching from *Synechococcus* to *Prochlorococcus* dominance in TR1 and TR2, respectively, is consistent with previous studies: mesotrophic water communities dominated by *Synechococcus* and picoeukaryotes versus oligotrophic water communities dominated by *Prochlorococcus* (Zubkov et al., 1998).

## Conclusion

Our data provide an image of pico and nanoplankton abundances in Southwest Atlantic waters along the Brazilian Bight. The different water masses played important roles to structure of pico and nanoplankton communities. In TR1, the uplifting of nutrient rich waters seemed to induce an abundance increase in *Synechococcus*, pico- and nanophytoeukaryotes populations near the continental slope. In contrast, the most striking feature observed in TR2 was the dominance of *Prochlorococcus* throughout the oligotrophic Tropical Water. Despite the differences observed in the top of the water column, autotrophic picoeukaryotes dominated the carbon stock near DCM in both transects, possibly linked to the proximity with the nutrient-rich SACW associated with a higher tolerance to lower light levels within this group.

##  Supplemental Information

10.7717/peerj.2587/supp-1 Figure S1 Example of flow cytometry gating patterns used to discriminate the different pico- and nanoplanktonic populations (A) Side scatter *versus* chlorophyll-a and (B) phycoerythrin *versus* chlorophyll-a: *Prochlorococcus* (pink), *Synechococcus* (green), picoeukaryotes (blue) and nanoeukaryotes (orange); (C) side scatter *versus* DNA fluorescence: heterotrophic bacteria (yellow). Calibration beads are marked in black.

10.7717/peerj.2587/supp-2 Figure S2 Surface distributions of temperature (T °C) (A), salinity (B), fluorescence (RFU) (C), nitrates (µM) (D) and phosphates (µM) (E)

10.7717/peerj.2587/supp-3 Figure S3 Surface distributions of total heterotrophic bacteria (A), *Prochlorococcus* (B), *Synechococcus* (C), picoeukaryotes (D) and nanoeukaryotes (E) in cells mL^−1^

10.7717/peerj.2587/supp-4 Figure S4 Vertical distributions of biotic data Vertical distributions (from the top) of: total autotrophic biomass (µgC L^−1^), relative contribution to total biomass (in percentage) of total heterotrophic bacteria, and relative contribution to autotrophic biomass (in percentage) of *Prochlorococcus*, *Synechococcus* and picoeukaryotes, for TR1 (A–E, right column) and TR2 (F–J, left column); numbers indicate sampling stations; dashed white lines represent the boundary between water masses; TW: Tropical Water; SACW: South Atlantic Central Water; CW: Coastal Water.

10.7717/peerj.2587/supp-5 Figure S5 Surface distributions of biotic data Surface distributions of total autotrophic biomass (µgC.L^−1^) (A), relative contribution to total biomass (in percentage) of total heterotrophic bacteria (B), relative contribution to autotrophic biomass (in percentage) of *Prochlorococcus* (C), *Synechococcus* (D) and picoeukaryotes (E).

## Additional Information and Declarations

### Data Availability

The following information was supplied regarding data availability: Ribeiro, Catherine; Lopes dos Santos, Adriana; Marie, Dominique; Pellizari, Vivian Helena; Pereira Brandini, Frederico; Vaulot, Daniel (2016): Pico and Nanoplankton Abundance and Carbon Stocks along the Brazilian Bight. PeerJ. Supplementary material. Table S1. Figshare: https://dx.doi.org/10.6084/m9.figshare.3492098.v2.
