# Supplementary figures and images for: Pico and nanoplankton abundance and carbon stocks along the Brazilian Bight
Source: PeerJ. 2016 Nov 10;4:e2587. doi: 10.7717/peerj.2587 (PMC5111892; doi:10.7717/peerj.2587)

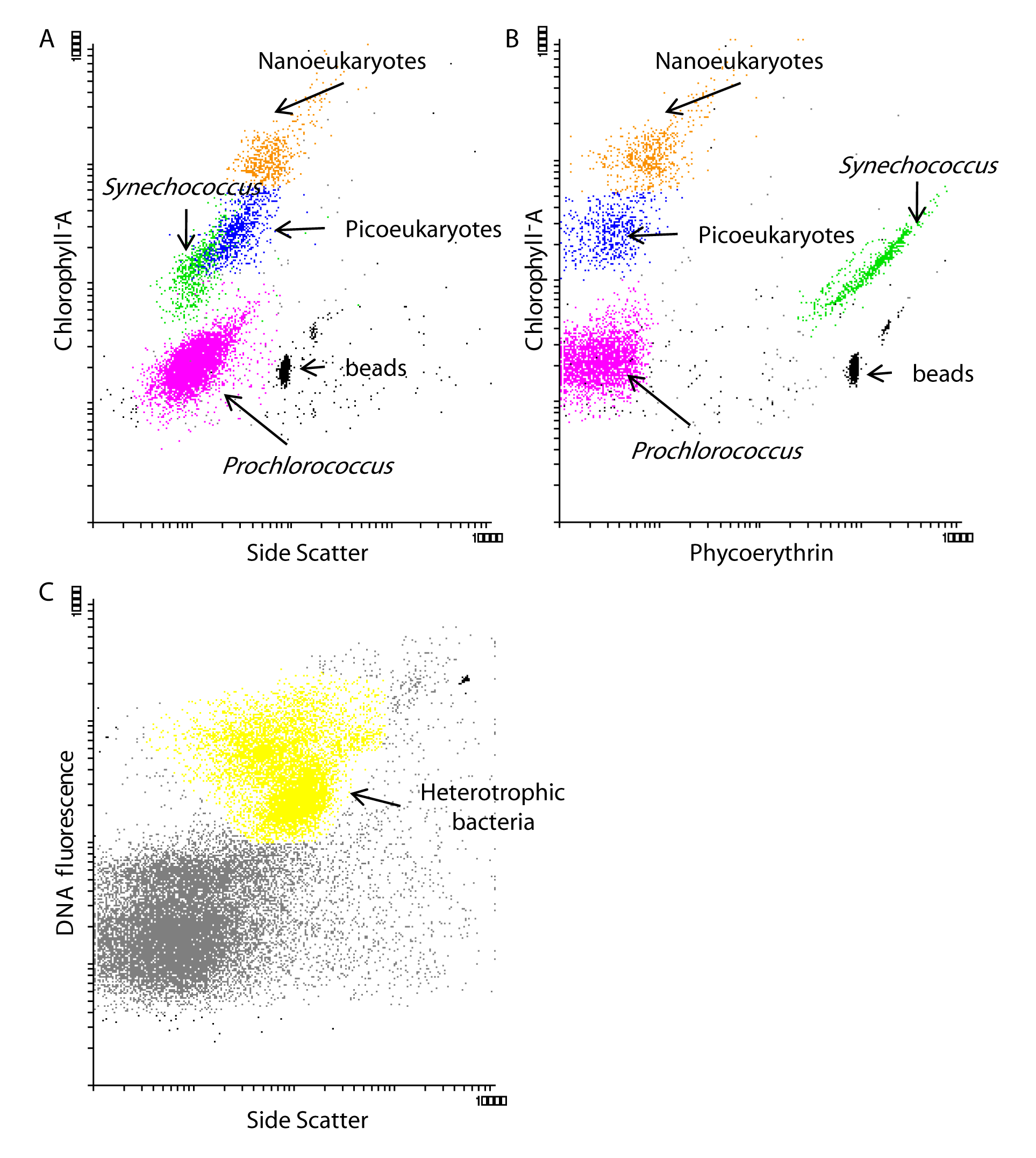

Supplement: Figure S1 — (A) Side scatter versus chlorophyll-a and (B) phycoerythrin versus chlorophyll-a: Prochlorococcus (pink), Synechococcus (green), picoeukaryotes (blue) and nanoeukaryotes (orange); (C) side scatter versus DNA fluorescence: heterotrophic bacteria (yellow). Calibration beads are marked in black. [file peerj-04-2587-s001.png]

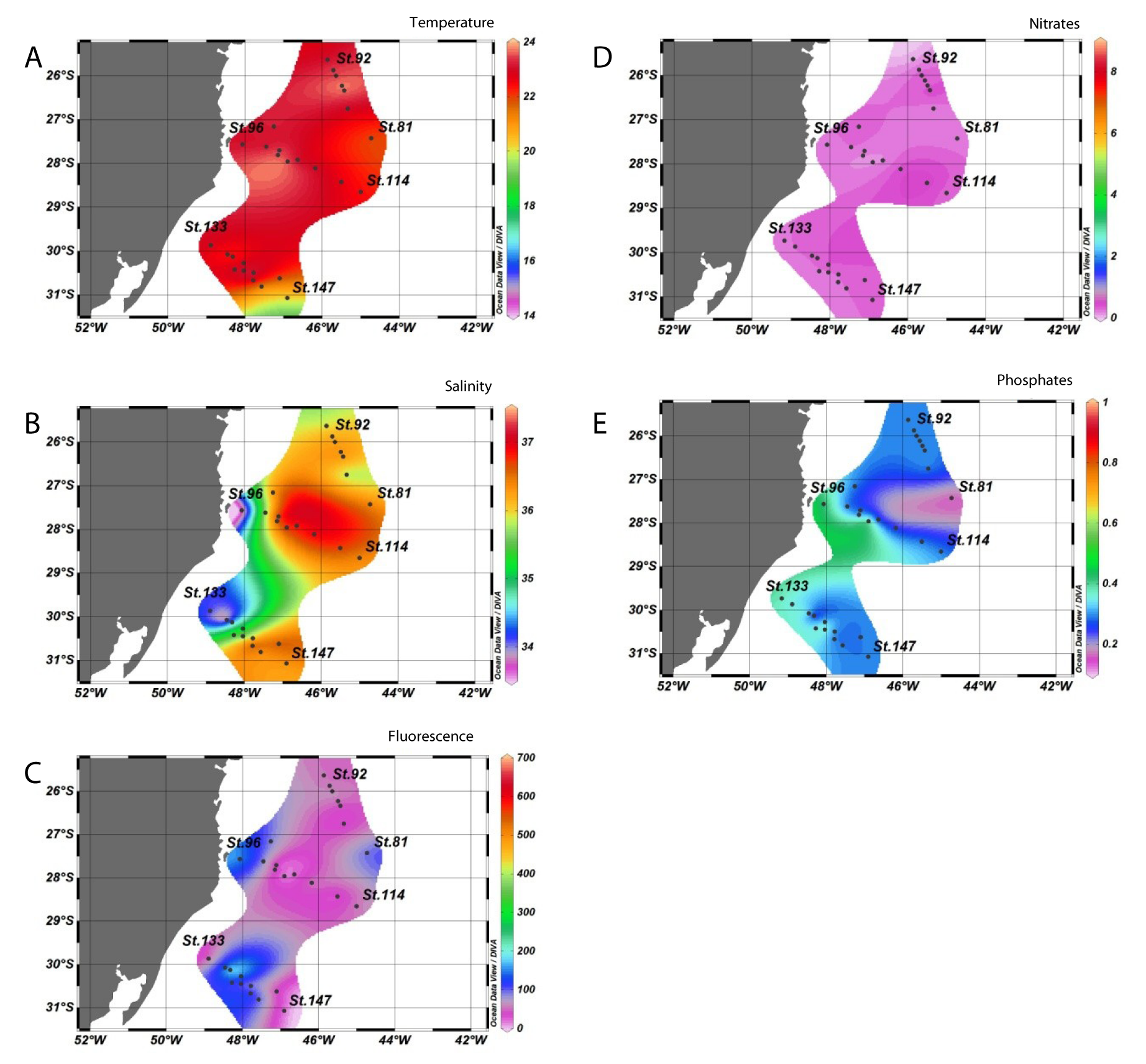

Supplement: Figure S2 [file peerj-04-2587-s002.png]

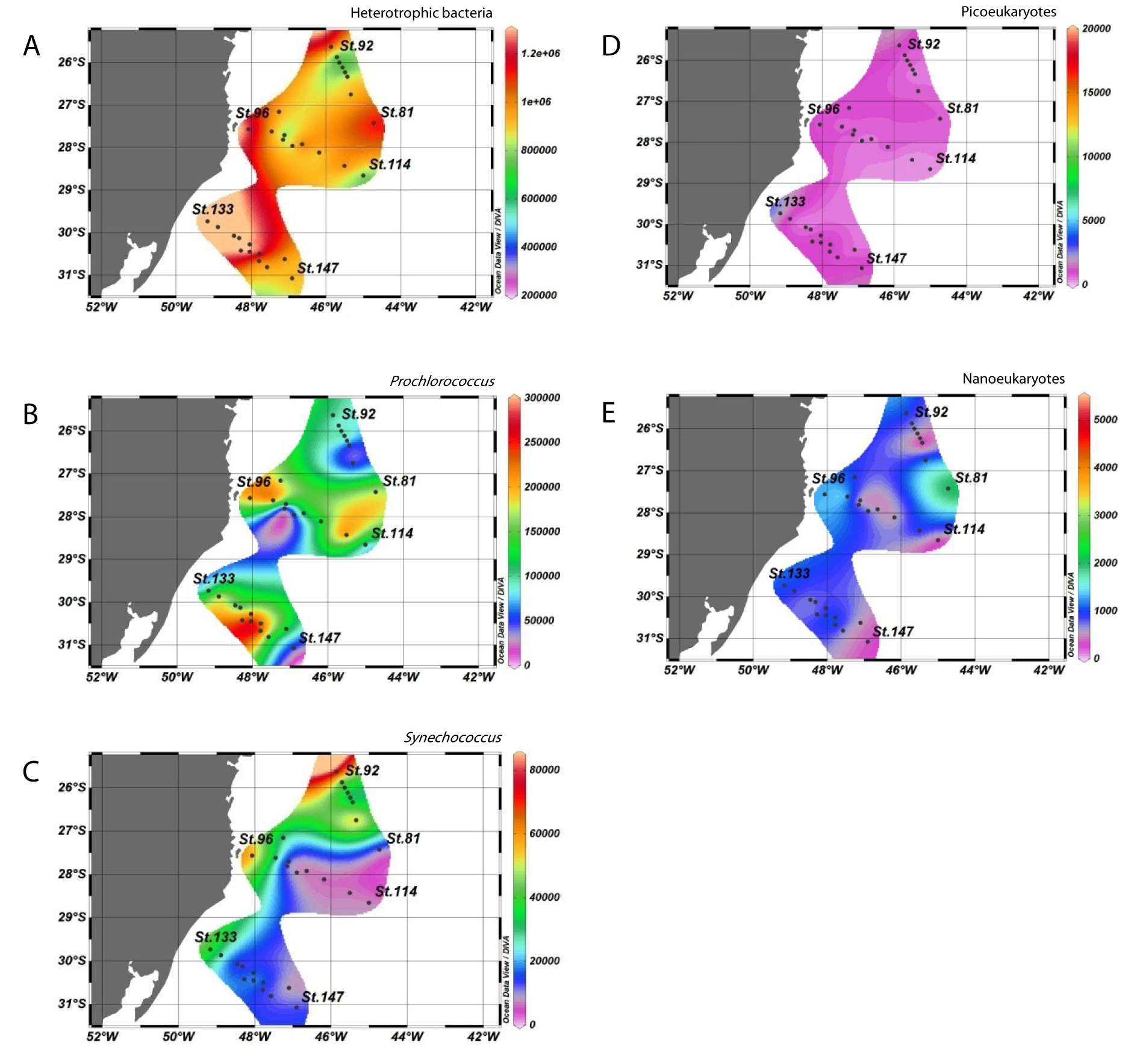

Supplement: Figure S3 [file peerj-04-2587-s003.png]

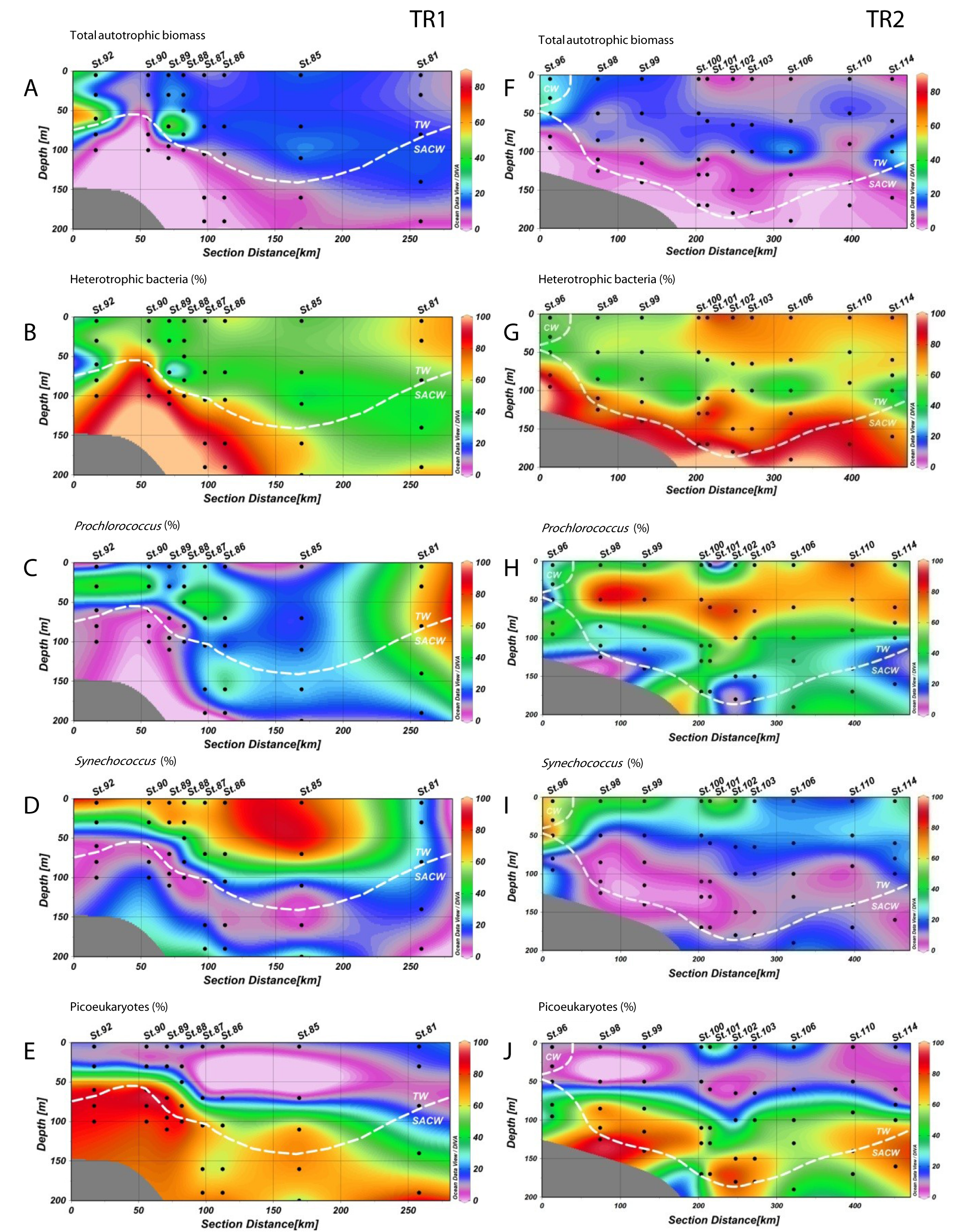

Supplement: Figure S4 — Vertical distributions (from the top) of: total autotrophic biomass (µgC L−1), relative contribution to total biomass (in percentage) of total heterotrophic bacteria, and relative contribution to autotrophic biomass (in percentage) of Prochlorococcus, Synechococcus and picoeukaryotes, for TR1 (A–E, right column) and TR2 (F–J, left column); numbers indicate sampling stations; dashed white lines represent the boundary between water masses; TW: Tropical Water; SACW: South Atlantic Central Water; CW: Coastal Water. [file peerj-04-2587-s004.png]

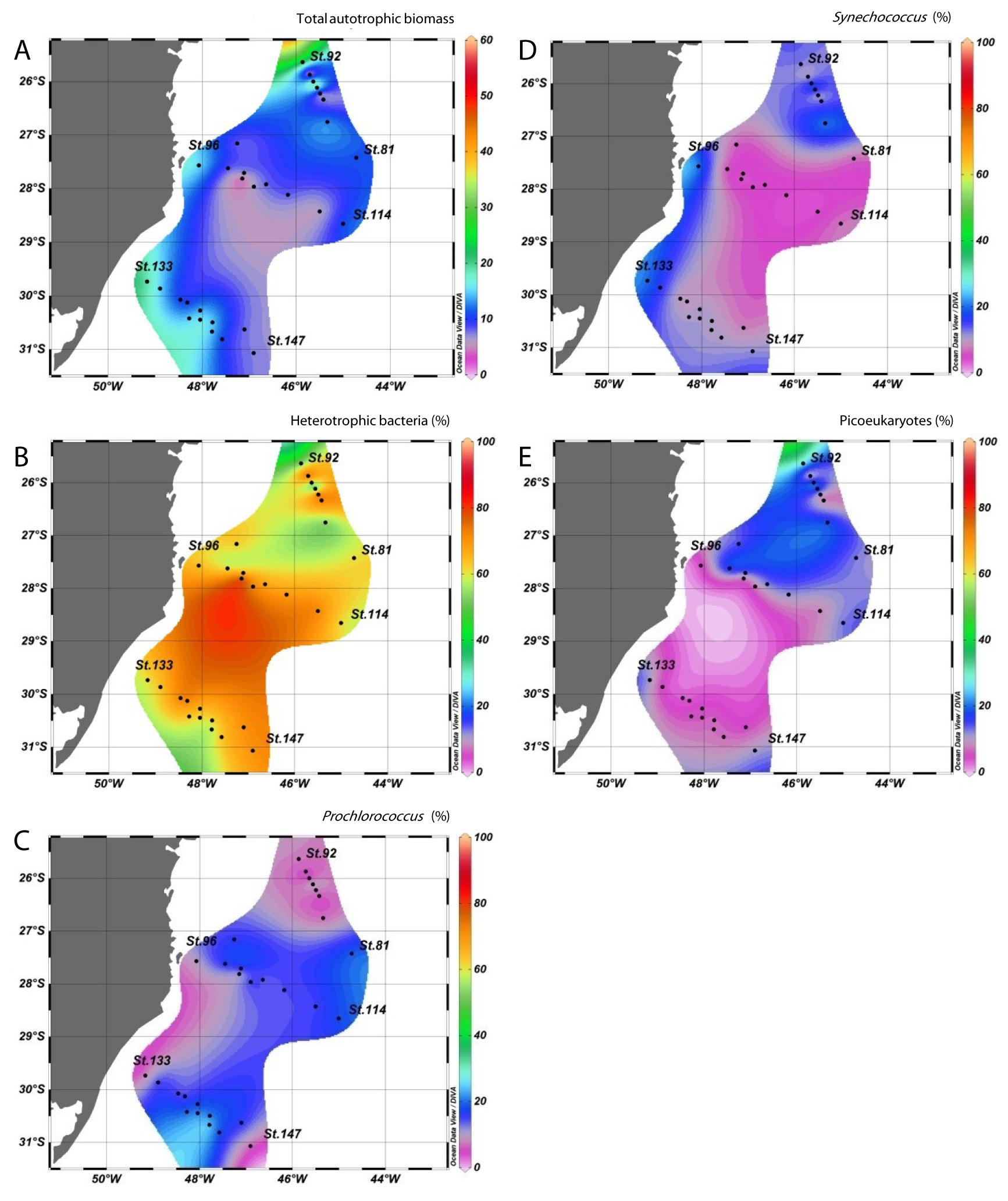

Supplement: Figure S5 — Surface distributions of total autotrophic biomass (µgC.L−1) (A), relative contribution to total biomass (in percentage) of total heterotrophic bacteria (B), relative contribution to autotrophic biomass (in percentage) of Prochlorococcus (C), Synechococcus (D) and picoeukaryotes (E). [file peerj-04-2587-s005.png]
